# Critical genes in genitourinary embryogenesis are related to the development of adult hydrocele

**DOI:** 10.1038/s41598-024-81187-3

**Published:** 2024-12-05

**Authors:** Jeffrey L. Roberson, Christopher J Neylan, Renae Judy, Venexia Walker, Philip S. Tsao, Scott M. Damrauer, Lillias H. Maguire

**Affiliations:** 1 Department of Surgery, Perelman School of Medicine, Philadelphia, PA USA; 2 Medical Research Council Integrative Epidemiology Unit, University of Bristol, Bristol, UK; 3 VA Palo Alto Epidemiology Research and Information Center for Genomics, VA Palo Alto Health Care System, Palo Alto, CA USA; 4 Department of Medicine, Stanford University School of Medicine, Stanford, CA USA; 5 Stanford Cardiovascular Institute, Stanford University School of Medicine, Stanford, CA USA; 6 Corporal Michael J. Crescenz Memorial Veterans Affairs Medical Center, Philadelphia, PA USA; 7 Department of Genetics, University of Pennsylvania Perelman School of Medicine, Philadelphia, PA USA; 8 Division of Colon and Rectal Surgery, Department of Surgery, Hospital of the University of Pennsylvania, 3400 Spruce Street, 4 Silverstein, Philadelphia, PA 19104 USA

**Keywords:** Hydrocele, GWAS, Herniorrhaphy, Disease genetics, Urological manifestations

## Abstract

Despite being a common urologic disorder with potentially complicated sequela, the genetic background of adult hydrocele has not previously been described. We performed a multi-population genome-wide association study of 363,460 men in the United Kingdom BioBank and FinnGen cohorts. We identified 6,548 adult men with hydrocele. We analyzed common variants (minor allele frequency > 0.01) associated with hydrocele and set the threshold for genome-wide significance at *p* < 5 × 10^− 8^. Meta-analysis of genome-wide association studies identified 7 genome-wide significant loci which mapped to 24 genes. Multiple gene prioritization strategies highlighted PAX8, *INHBB*,* AMHR2*, and *SHH*, all known to be critical to genitourinary embryogenesis and associated with Mendelian genitourinary syndromes and model organism phenotypes. Identified loci affect gene expression in genitourinary structures and are associated with multiple markers of renal function. These common variants in genes critical for genitourinary embryogenesis are associated with adult hydrocele, suggesting these genes may maintain normal scrotal anatomy in adults. This large study of nearly 400,000 men is the first genomic study of idiopathic hydrocele and defines our current understanding of the genetic background of this common condition.

**Supplementary Information:**

The online version contains supplementary material available at 10.1038/s41598-024-81187-3.

## Introduction

Hydrocele, an abnormal collection of serous fluid surrounding the testicle, is a common urologic condition and the most common cause of scrotal swelling. From an etiologic standpoint, hydroceles can be grouped into congenital and acquired. Congenital hydroceles are due to failed obliteration of the processus vaginalis, an extension of the peritoneal cavity that surrounds the testes as they descend into the scrotum. The proximal aspect of this peritoneum typically undergoes programmed cell death, and the distal component becomes the tunica vaginalis enveloping the testes^1–3^. The vast majority of congenital hydroceles resolve in early childhood. Acquired hydroceles develop in adolescence and adulthood and are thought to be due to an imbalance of secretion and absorption of fluid in the tunica vaginalis, potentially due to lymphatic obstruction. In temperate, high-income countries, most hydroceles present in middle age and are idiopathic, although they may occur secondary to infection, trauma, inguinal or testicular surgery, malignancy, or ascites^4^. In tropical low- and middle-income countries, however, by far the most common cause of secondary hydrocele is filariasis with *Wuchereria bancrofti*^3^.

Research on adult hydrocele is largely focused on the indications and techniques for medical and surgical management^5–10^. Despite the common nature of the condition, there is little etiologic research on inherent risk factors or heritability. By contrast, family studies, twin studies, and genome-wide association studies (GWAS) have been performed for inguinal hernia, another inguino-scrotal condition with a congenital/acquired etiologic dichotomy, involvement of biology of the processus vaginalis, and significant diagnostic overlap with hydrocele. These studies have shed light on the biologic basis of inguinal hernia and highlight the potential of genomic analyses to increase understanding of common diseases.

In this study, we perform a novel investigation into the contribution of the genetic background of persistent adult hydrocele. We study 6,548 adult men with hydrocele, identifying genetic loci associated with hydrocele, and putatively mapping these loci to genes that imply a fundamentally different genetic architecture for hydrocele than its closely clinically related condition inguinal hernia.

## Methods

### Overview

We performed a combined analysis of two large, genotyped cohorts, identified variants associated with clinically detected hydrocele based on diagnosis codes, and physically mapped those variants to discrete loci in the human genome. We identified 24 genes present at these 7 loci. To prioritize those genes at each locus we assessed for overlap with regions of the genome that effect gene expression (expression quantitative trait loci [eQTLs]) and association of those variants and genes with human or model organism traits relevant to hydrocele.

### Study populations, genotyping, and phenotyping

#### United Kingdom Biobank (UKBB)

In UKBB, individuals aged 45–69 years old from across the United Kingdom were recruited for genotyping, survey-based phenotyping, and linkage to the electronic health record (EHR)^11^. Genome-wide genotyping was performed on all UK Biobank participants using the UK Biobank Axiom Array. Approximately 850,000 variants were directly measured, with > 90 million variants imputed using the Haplotype Reference Consortium and UK10K + 1000 Genomes reference panels. Individuals were designated as having a hydrocele if the ICD10 code N43 was present in their self-reported illness codes or EHR-based ICD codes.

#### FinnGen

FinnGen consists historical samples collected by the National Institute for Health and Welfare and additional prospectively collected from hospital biobanks^12^. Unlike the UKBB which specifically recruited middle-aged individuals, FinnGen recruited participants from across the adult age range. Participating individuals consent to linkage of genome-wide genotyping with nationwide registers of longitudinal health data. FinnGen participants are genotyped on a custom array. The current array (v2) contains 723,376 probesets for 664,510 markers. All GWAS data is imputed against a Finnish population specific whole genome sequence (WGS) backbone. Patients with hydrocele were identified through administrative coding using the ICD-10 code N43. LiftOver^13^ was used to map genome positions from hg38 to hg19.

### Combined analysis of GWAS

Summary statistics were obtained from all male participants in FinnGen and UKBB for combined analysis. These groups were combined GWAS. Only SNPs with mean allele frequency (MAF) > 1% in these studies were used. Combined analysis was performed using a fixed-effects model via METAL^14^ with inverse-variance weighting of log odds ratios. Variants were considered genome-wide significant if they passed the conventional P-value threshold of 5 × 10^− 8^.

Variants were physically mapped to independent loci using both DEPICT and FUMA^15^. In FUMA, genome-wide significant SNPs independent from each other at *r*^*2*^ < 0.5 were defined as independent SNPs and positionally mapped to genomic loci. Lead SNPs were defined as independent from each other at *r*^*2*^ < 0.1. The 1000 Genomes Phase 3 reference panel was used to compute *r*^2^. A genomic locus was defined as a ± 250 kb interval in which one or more identified SNPs reached genome-wide significance. Physically close loci (± 250 kb) were merged into one locus. In DEPICT, genes are mapped to loci if they resided within, or overlap, boundaries defined by the most distal SNPs in either direction with LD r^2^ > 0.5 to the given lead SNP. If no genes were within the locus defined by r^2^ > 0.5, the gene nearest to the given lead SNP was included. In addition to physical mapping, expression quantitative trait (eQTL) mapping was performed. Independent SNPs and those in linkage disequilibrium with them were analyzed for overlap with cis-eQTLs (genes at less than 1 Mb distance) in the following GTEX version 8 tissues: prostate, testis, kidney cortex, and adrenal gland.

#### Genetic colocalization

In cis-eQTL-overlapping loci, we assessed for the colocalization of genetic association signals between single tissue cis-eQTLs and hydrocele-associated variants using COLOC^27^. This analysis aims to answer whether the regions associated with a particular gene’s expression and hydrocele not only overlap, but also share a common causal variant. We investigated ± 250 kb windows surrounding the loci. Evidence of colocalization at a causal variant was defined as a conditional probability of localization based on high Bayesian posterior probabilities of colocalization (i.e. PP4/PP4 + PP3) > 0.75.

#### Genetic correlation

We estimated genetic correlation of hydrocele with inguinal hernia (ICD10 K40) in UKBB participants using LD Score Regression (LDSC)^28^ and summary statistics obtained from the Neale lab (https://nealelab.github.io/UKBB_ldsc/downloads.html). LDSC estimates cross-trait correlation without requiring individual-level data and without bias from overlapping samples. This method uses all SNPs (not only genome-side significant ones) and looks for cross-trait correlations in effect sizes for each SNP.

#### Associated traits and gene expression

We used the Open GWAS Project^16^ to query over 42,000 previously performed studies for prior associations with hydrocele-associated SNPs at *p* < 5 × 10^− 8^. We queried ClinVar^17^ for short (< 50 kb) pathogenic variants in our mapped genes associated with human diseases related renal, genitourinary, or reproductive phenotypes. We queried the Mouse Genome Database Project^18^ to identify knock-out mice in our genes of interest with phenotypes relevant to renal, genitourinary, or reproductive biology. Additionally, we queried the Open GWAS Project for associations our SNPs with potential causes of secondary hydrocele such as orchitis/epididymitis, testicular cancer, and testicular torsion.

## Results

**Fig. 1:**
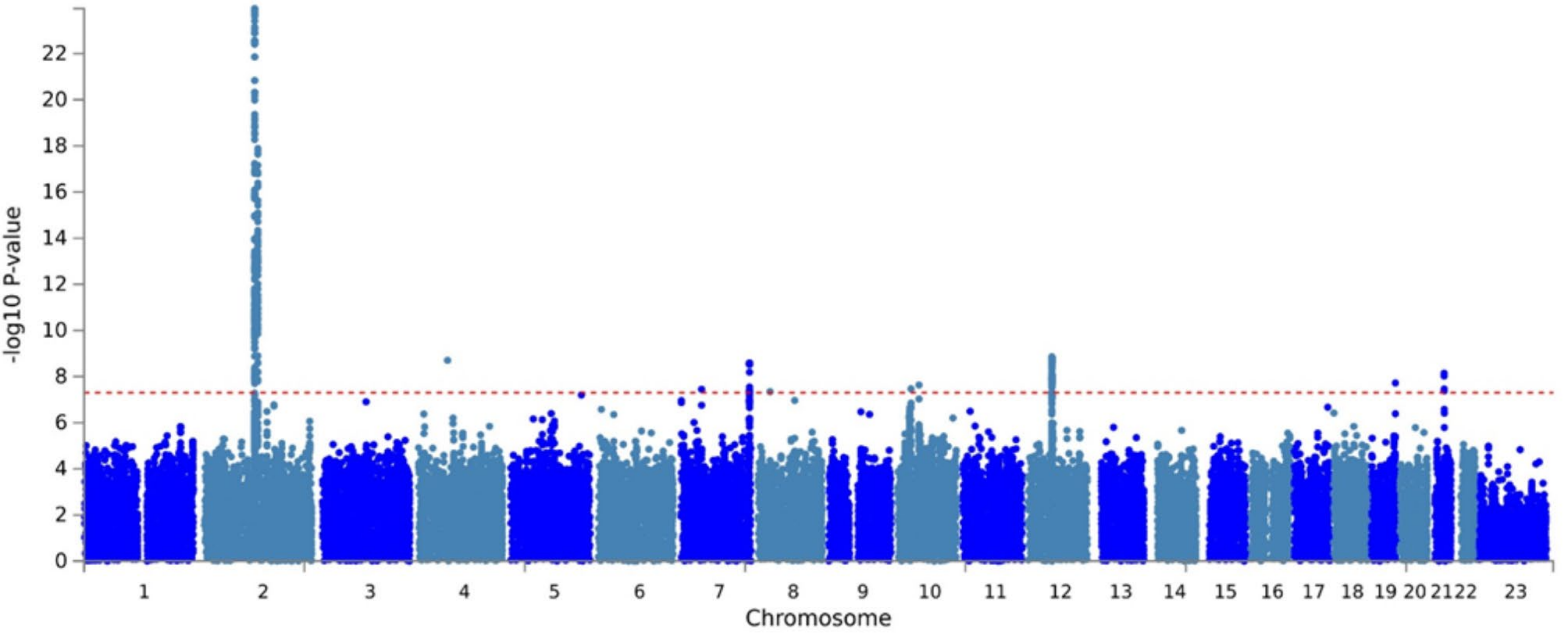
Manhattan Plot of variants associated with hydrocele. Red line indicates threshold for genome-wide significance (*p* < 5 × 10^− 8^).

We identified 6,548 men with hydrocele among 363,640 male participants (a total of 211,840 from UKBB and 151,800 from FinnGen). Following combined analysis, a total of 25 independent variants at 7 genetic loci met genome-wide significance threshold (Table 1; Fig. 1). The vast majority of variants were in noncoding regions, but four independent SNPs were in linkage disequilibrium with coding variants in *ATF7* and *SP1.*

We evaluated all previously reported associations with the independent significant SNPs at each hydrocele-reported locus using OpenGWAS (Supplementary Table 1). This analysis revealed multiple associations, but notably we identified multiple serum markers of renal function in two loci including glomerular filtration rate (GFR), calcium, cystatin C levels, urate, and creatinine. Some of these associations were noted in BioBank Japan, indicating relevance across multiple populations. Additionally, we identified associations with genitourinary cancers including mucinous ovarian carcinoma and cervical dysplasia. None of the SNPs were associated with inguinal hernia. None of the SNPs were associated with potential causes of secondary hydrocele.

**Table 1:** Lead variants associated with hydrocele.

Locus	Chr	Pos: Start	Pos: End	Lead SNP	*P*	Beta	SE	Nearest Gene
1	2	113,984,033	114,064,671	rs3748916	1.12E-24	-0.2484	0.0242	*PAX8*
2	2	121,125,878	121,134,595	2:121125878:G: T	1.31E-18	0.0026	0.0003	*INHBB*
3	7	155,653,078	155,656,235	rs1616634	2.56E-09	0.1409	0.0236	*SHH*
4	8	27,454,089	27,454,089	rs2741346	4.50E-08	0.0055	0.001	*CLU*
5	10	31,395,761	31,411,355	rs1087056	3.40E-08	0.0017	0.0003	*ZNF438*
6	10	49,518,772	49,530,358	rs111302388	2.32E-08	0.0021	0.0004	*MAPK8*
7	12	53,814,380	54,072,180	rs11170549	1.41E-09	0.0023	0.0004	*AMHR2*

*Chr* chromosome, *Pos* position, *SNP* single nucleotide polymorphism, *P* p-value, *SE* standard error of beta effect.

**Fig. 2:**
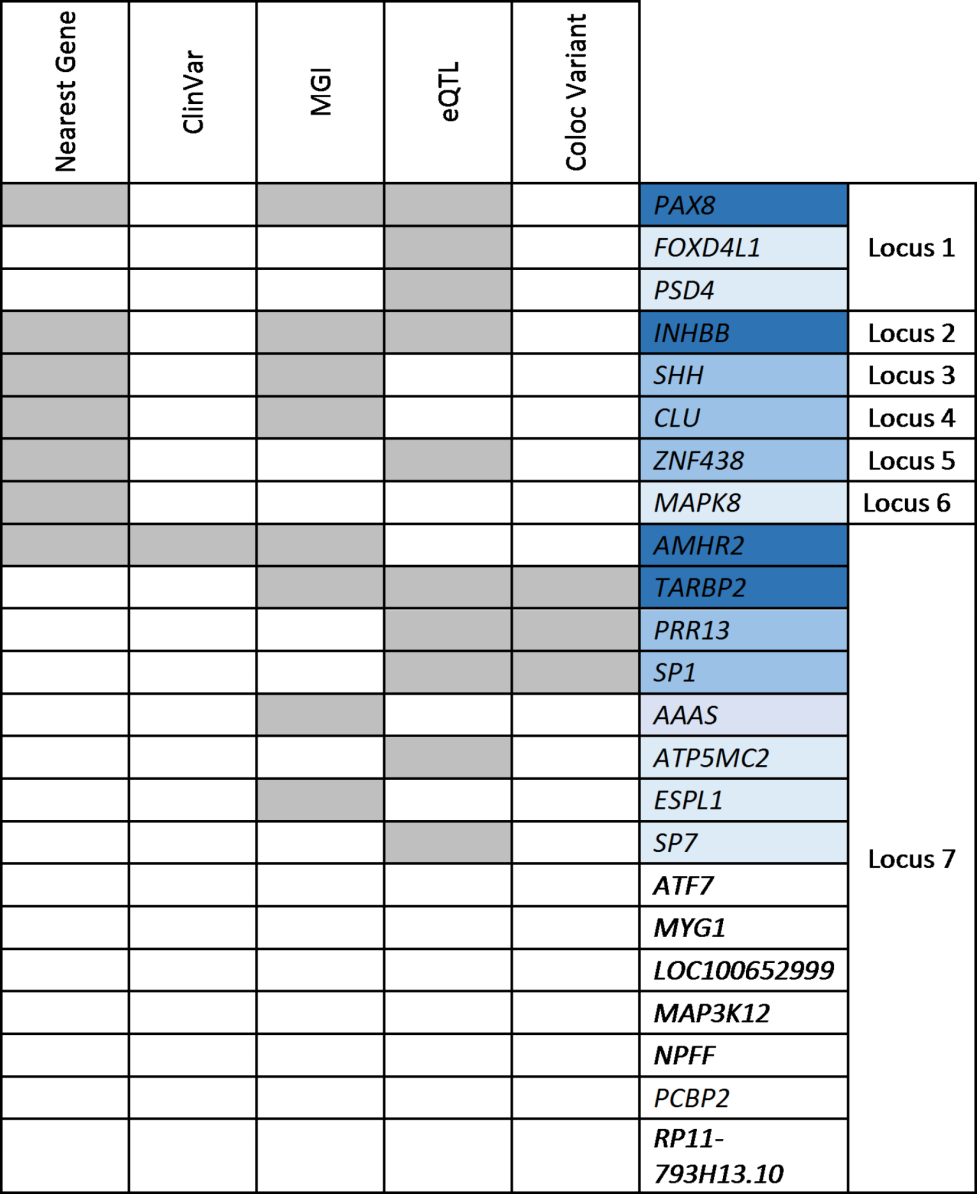
Gene Prioritization Results. Colors of gene symbols indicate increasing levels of evidence. *eQTL* expression quantitative trait locus, *MGI* Mouse Genome Informatics relevant knockout mouse phenotype, *ClinVar* relevant human disease, *Coloc variant* shared variant for hydrocele and eQTL in relevant tissue.

There were 24 genes physically located close to the seven loci, including one highly polygenic locus on chromosome 12. SNP to gene identification was slightly different between FUMA and DEPICT. We included all genes identified by either program. We then applied five gene prioritization strategies: nearest physical gene, overlap of cis-eQTL in a relevant human tissue, colocalization in a single variant of association with both hydrocele and cis-eQTL, and prior association with a mouse or human renal, genitourinary, or reproductive phenotype (Supplementary Tables 2–4). Three genes, *INHBB*,* PAX8*,* TARBP2*, and *AMHR2*, met three criteria and six others *CLU*,* SHH*,* PRR13*,* SP1*, and *ZNF438* met two (Fig. 2).

LD Score Regression analysis did not reveal significant genetic correlation between hydrocele and inguinal hernia (*p* = 0.38, rg = 0.11).

## Discussion

Despite the commonality of hydrocele, there is little etiologic research into this condition. In this study, we identified 7 loci with hydrocele in over 6000 men. We prioritized four genes, *PAX8*, *INHBB*, *TARBP2*, and *AMHR2*, that met three or more gene prioritization criteria. *PAX8*,* INHBB*, and *AMHR2* have well-described roles in genitourinary development and are associated with severe human and/or model organism phenotypes. However, a role for them in the development of a common adult trait is novel.

*PAX8* is a transcription factor critical for normal human development of multiple organs including the kidney and the epithelial layers of the Mullerian tract^19,20^. Multiple families with deleterious *PAX8* mutations have been described with congenital genitourinary anomalies including hydrocele, horseshoe kidney, cryptorchidism, and renal agenesis in the context of congenital hypothyroidism. *PAX8* expression persists in adult men in the seminal vesicles and epididymis^20,21^. Variants in our study were in an eQTL for *PAX8*,* PAX8-AS1*, and *PSD4* expression testis, uterus, vagina, adrenal gland, and prostate. Altered expression of *PAX8* is described in Wilms tumor, ovarian, uterine, renal, and prostatic malignancies, making it a clinically useful tumor marker^22,23^. In mice, *PAX8* knockouts demonstrate male and female infertility and multiple genitourinary and reproductive anomalies.

*AMHR2* encodes the anti-Mullerian hormone receptor which is critical to male sexual development. In male fetal development, *AMHR2* initiates the regression of Mullerian ducts in response to Sertoli cell secretion of anti-Mullerian hormone (AMH). Loss of function mutations in either AMH or the receptor anti-Mullerian hormone receptor type II (*AMHR2*) leads to persistence of Mullerian duct derivatives in individuals with XY chromosome composition^24–26^. Knockout mice display a similar phenotype.

*INHBB* encodes the β_B_ subunit of inhibin B, a testicular glycopeptide hormone which inhibits follicular stimulating hormone. In adult men *INHBB* level is good marker of spermatogenesis, correlating with sperm count and testis volume. The role of *INHBB* in testicular physiology is under investigation. Men with polymorphisms in *INHBB* anatomically normal genitalia with infertility. However, *INHBB* knockout mice have normal fertility and altered testis cell composition. Specific to anatomic development of the testicles, *INHBB* may influence development of the testicular vasculature in males^27,28^.

*TARBP2* is a member of the Dicer1 complex which is critical in processing microRNAs. microRNAs are increasingly appreciated to play a role in nephron development. Mutations in this complex, including in *TARBP2*, lead to Dicer1 Syndrome which includes multiple rare malignancies including cystic nephromas and Sertoli-Leydig cell tumors. Additionally, *TARBP2* mutations are found in Wilms tumor.

Finally, *SHH*, although meeting fewer gene prioritization criteria, is a key signaling factor in both genitourinary development and cancer. In humans, *SHH* mutations result in congenital urinary tract malformations and *SHH-*knockout mice demonstrate multiple genitourinary anomalies. More recently, aberrations to the *SHH* pathway have been implicated in cancers of the genitourinary tract in both men and women^29^ including clear cell renal^30^, urinary bladder^31^, ovarian^32^, cervical^33^, and prostate cancer^31^.

Several genome-wide significant variants associated with hydrocele have been previously associated with markers of renal function and genitourinary cancers. However, despite the anatomic and clinical overlap, none of the loci or mapped hydrocele genes from our study have previously been associated with inguinal hernia, suggesting a distinct genetic architecture for these two conditions. LD Score regression did not reveal significant genetic correlation. Inguinal hernia is largely associated with variants encoding key connective tissue genes^34^, whereas our study reveals hydrocele to be associated with genes key for normal anatomic and hormonal sexual development.

One prior GWAS of hydrocele was performed in a Ghanian population of men afflicted with filarial disease^35^. This study identified two variants at the HLA locus, typical for an infectious pathology and in-keeping with prior data suggesting host factors effect disease phenotype. We did not replicate these loci in this study, which is consistent with the clinical appreciation of these conditions as two fundamentally different etiologies.

Our study should be considered in the context of its limitations. In particular, this GWAS is limited to high income countries, excluding the vast majority of global patients with hydrocele, whose disease is attributable to filariasis. Second, the de-identified nature of the data makes it impossible to distinguish between congenital and acquired hydrocele. Given that enrollment in all the study populations is limited to adults, it is likely that the majority are acquired and, in these geographic regions, idiopathic, but we are unable to determine what proportion are due to sequelae of infection, surgery, or ascites. However, although our phenotype may have included secondary hydroceles, none of our SNPs associated with common causes of secondary hydrocele, suggesting they are not confounded by association with another phenotype. Additionally, it is possible that some hydroceles were misdiagnosed and represent an alternate or co-esiting pathology, such as inguinal hernia. Our eQTL analysis may be limited by the collected tissue types which, although including multiple genitourinary organs did not include inguinal-scrotal connective tissues, which may be relevant to the pathogenesis of hydrocele. Finally, it is likely that many patients in the study populations have a hydrocele that has not become clinically relevant, leading to inclusion of participants with hydrocele in the “control” group, although in general this would bias the analysis toward the null.

It is too early to predict the translational potential of improved understanding of the genetic background of hydrocele. However, novel therapeutic targets have high potential in any disease in which the treatment is purely surgical, recurrence is relatively common, and prevention, particularly of its complicating other procedures, is highly desirable. Some hydrocele-associated genes and related compounds have established or emerging clinical roles in genitourinary disease. Anti-Mullerian Hormone (AMH), the ligand of *AMHR2*, and INHBB are biomarkers for Sertoli cell function^36^. AMH has an emerging role as a female contraceptive^37^ and INHBB-inhibitor follistatin, itself critical in normal testicular function, shows early renal-protective effects^38,39^. These data suggest these genes have translational potential and our study, though early, suggests that potential could be extended to hydrocele.

## Conclusion

We identified 24 genes associated with hydrocele in a population of nearly 400,000 men. Gene prioritization identified *PAX8*, *INHBB*, *AMHR2*, and *SHH*, genes with known to be pivotal roles into genitourinary development and *TARBP2* which is implicated in renal tumors. The association of common variants in these genes with adult hydrocele suggests potential novel roles for these genes in maintaining normal scrotal anatomy in adults.

## Electronic supplementary material

Below is the link to the electronic supplementary material.

Supplementary Material 1

Supplementary Material 2

Supplementary Material 3

## Data Availability

FinnGen and UKBB summary statistics are available to all investigators at https://www.finngen.fi/en/access_results and https://nealelab.github.io/UKBB_ldsc/downloads.html#full_gwas_results respectively. GTEX tissue expression data are available at https://gtexportal.org/home/downloads/adult-gtex/overview. All software packages referenced within the paper are open source.
